# The Role of Unemployment, Financial Hardship, and Economic Recession on Suicidal Behaviors and Interventions to Mitigate Their Impact: A Review

**DOI:** 10.3389/fpubh.2022.907052

**Published:** 2022-07-06

**Authors:** Sharna Mathieu, Alice Treloar, Jacinta Hawgood, Victoria Ross, Kairi Kõlves

**Affiliations:** Australian Institute for Suicide Research and Prevention and WHO Collaborating Centre for Research and Training in Suicide Prevention, School of Applied Psychology, Griffith University, Brisbane, QLD, Australia

**Keywords:** suicide, self-harm, unemployment, financial hardship, economic recession, welfare benefits, COVID-19

## Abstract

Understanding the social determinants and risk factors for suicidal behaviors underlies the development of effective suicide prevention interventions. This review focused on recently published literature (2010 onwards), with the aim to determine the role of economic factors (at the individual and population level) on suicidal behaviors and ideation as well as the effectiveness of interventions addressing these factors in reducing suicidal behaviors and ideation. Where available, literature examining the economic impact of COVID-19 was highlighted. Economic recession and unemployment are associated with increased risk of suicidal behavior at the population and individual level. Additionally, personal financial problems such as debt and financial strain are associated with increased risk of suicidal behavior and ideation at the individual level. Regarding interventions, unemployment benefits, employment protection legislation, higher minimum wage and active labor market programs may reduce suicide at the population level. However, it is not clear what impact they have at the individual level, nor in relation to suicide attempts, self-harm, or suicidal ideation. There was a lack of evidence as to the effectiveness of financially focused suicide prevention interventions at either level. Current findings were contextualized within, and advance, prominent social theoretical models. Recommendations focused on future areas of research, including the unfolding economic impact of COVID-19, as well as the co-design and evaluation of tailored interventions and/or gatekeeper training for those in the financial and welfare sector, and enhanced early education aimed at increasing financial literacy in young people before onset or exacerbation of financial hardship.

## Introduction

Suicidal behaviors and ideation have an immense and far-reaching impact on people, communities, and healthcare systems around the world. According to the World Health Organization (1), ~703,000 people died by suicide in 2019, representing an age-standardized rate of 9.0 per 100,000. The World Health Organization also estimate that for every suicide there are 20 or more suicide attempts (2). However, estimating the true prevalence of non-fatal suicidal behaviors and ideation is difficult given challenges with surveillance and an inability to capture the many individuals who do not seek or receive healthcare intervention for their suicide attempt, self-harm, or suicidal ideation (3).

Suicide is a complex, multifaceted issue with many interrelated and co-occurring biopsychosocial determinants at the individual and societal level (4). Understanding the risk and protective factors for suicidal behaviors (including suicide, suicide attempts and self-harm) and ideation is crucial to informing adequate prevention policies and developing effective interventions. Economic factors are critical and established social determinants of health and health equity, whereby those with escalating poverty and financial concerns experience ongoing and systemic issues with their health, including accessing adequate care (5). The detrimental effect of economic factors on mental health and suicide at the individual and societal level is increasingly recognized [e.g., (4, 6)].

Prominent economic factors at the individual level can include, among others, financial hardship (e.g., inability to repay debt), short- and long-term unemployment, underemployment (e.g., working less than desired or required due to economic reasons), overqualification, and job insecurity or precarious employment (7). At the population level (also referred to as aggregated, societal, or ecological level), macroeconomic factors most frequently include the overall unemployment rate, gross domestic product (GDP), and time periods of economic crisis/recession (8, 9). Each of these factors are greatly influenced by global and national economic events and policies, as well as fallout and response to environmental and social disasters (10). Indeed, economic factors at both the individual and societal level are intrinsically linked and mutually reinforcing.

**Figure 1:**
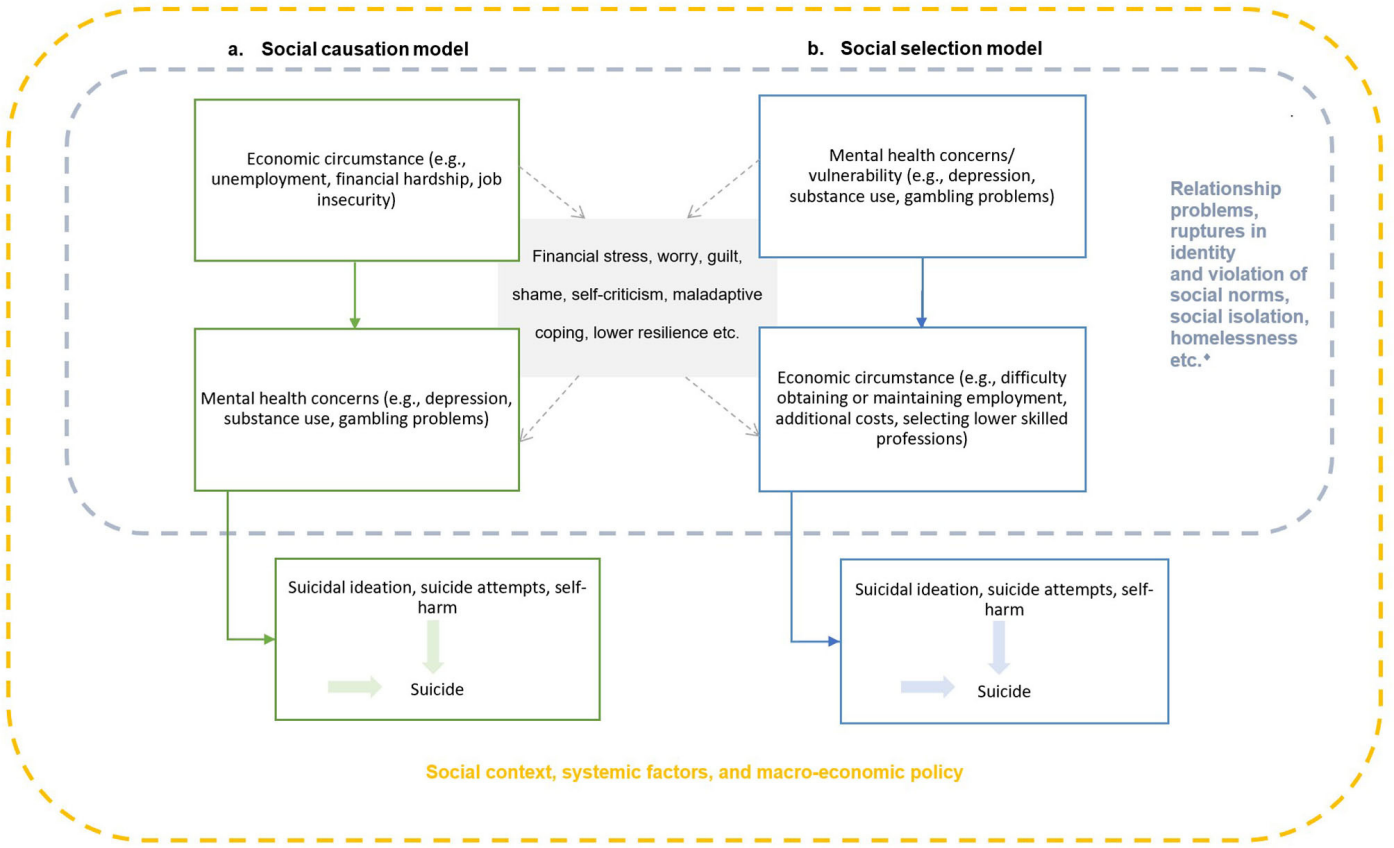
The social causation and social selection theoretical models that account for economic circumstance, mental health, and suicidal behavior. The diamond symbol correlates for suicidal behaviors that may influenced by economic factors and mental health through social causation or selection (may interact differently due to other socio-demographic variables such as gender).

Economic factors have also long been associated with increased risk of suicidal behaviors (8, 9). The literature describes two leading hypothetical models for how economic factors and mental health may influence suicidal behaviors and ideation. The “social causation” model suggests that economic circumstances (e.g., unemployment, job insecurity, financial hardship) result in substantial anxiety and stress (i.e., financial stress) and mental health problems, and ultimately suicidal behaviors. The “social selection” model, however, proposes that underlying (or vulnerability to) mental health problems increase the likelihood of insecure employment, job loss or financial insecurity through social drift (either directly, or indirectly via unfair work practices etc.) and in turn suicidal behaviors (see Figure 1) (6, 11, 12). Each proposed model is situated within the broader socio-economic context and likely relate differently across socio-demographic characteristics (e.g., gender, race). Adding to the complexity of the issue, mental health and economic factors may also be further associated with, or influenced by, other prominent risk factors for suicide such as homelessness, social exclusion, or relationship problems (7). Each model, therefore, is not necessarily mutually exclusive (6, 7), and regardless of direction both highlight the intensifying and cumulative role of economic factors in explaining a portion of the risk for suicidal behaviors and ideation.

Altogether, these associations have important implications for suicide prevention. There is a timely need for enhanced understanding of the role of economic factors at both the individual and aggregated level, such as unemployment, underemployment, job insecurity, and financial hardship on suicidal behaviors and ideation, particularly as a result of the ongoing COVID-19 pandemic. It is also crucial that the effectiveness of interventions addressing these factors at the societal and individual level is examined in relation to reductions in suicidal behaviors.

Therefore, the aim of the current review was to synthesize recently published literature (2010 onwards) to answer the following research questions:

1. What is the role of economic factors such as un/underemployment, financial hardship, financial wellbeing, job insecurity, and economic crisis on suicidal behaviors and ideation?
2. What available evidence is there for the effectiveness of interventions addressing economic factors in reducing suicidal behaviors and ideation?

A distinction was made between findings across both the individual and aggregate levels, and the impact of COVID-19 was highlighted where available. Key findings were discussed in relation to furthering theoretical understanding, and recommendations for future research, policy and practice are provided.

## Materials and Methods

Previous systematic reviews have focused on specific economic variables such as unemployment or combined various related, yet distinct economic factors utilized across the included studies (e.g., unemployment and job insecurity). However, given the co-occurring and compounding associations between economic factors and suicidal behaviors across multiple levels these reviews are often unable to provide a comprehensive overview and a balanced synthesis of knowledge across various research disciplines, high- and lower-income countries, and the spectrum of suicidal behaviors and ideation. Therefore, a selective review of the published literature was undertaken to provide insight into the current state of the field from a broader perspective. The review was not designed to be exhaustive, instead findings were used to provide advances in theoretical understanding (Figure 1) and generate ideas for future research and prevention.

### Databases and Search Terms

This review used purposive sampling to identify relevant articles from PubMed, Scopus, Google Scholar, as well as the reference lists of relevant studies, reviews, and meta-analyses. The search terms used included a combination of: suicid^*^, suicidal ideation, suicide attempt^*^, suicide thoughts, selfharm, self-harm, self-injur^*^, self injur^*^, prevent^*^, unemploy^*^, underemploy^*^, debt^*^, financial strain, job insecurity, financial hardship, job precarity, financial wellbeing, financial counseling, welfare policies, unemployment benefits, public health, mental health, active labor market programmes, unemployment benefits, unemployment protection, employment protection, unemployment insurance, unemployment compensation, social protection, income support, social security, labor market, labor market, upskill^*^, job upskil^*^, welfare. Searches were limited to those published from 2010 onwards and in the English language.

### Selection and Prioritization of Studies

The primary outcome measure in all peer-reviewed articles was suicidal behaviors and/or ideation. However, for interventions at the individual and aggregate level, secondary outcomes included mental health symptoms and wellbeing. While problematic gambling can be accompanied by significant financial strain (often concealed from, or at the detriment to, close relationships) and is associated with both depressive symptoms and suicidality [e.g., (13–15)], this was considered outside the scope and purpose of the current review. Therefore, any study with a primary focus on the relationship between gambling and suicidal behaviors or ideation and/or interventions addressing problematic gambling to prevent suicidal behaviors or ideation were excluded.

Studies were prioritized if they were a systematic review or meta-analysis. For empirical studies, those that included multiple countries and more than 1 year of data were prioritized, as were population-based data linkage studies, or those which examined novel variables, populations (e.g., low- and middle-income countries; LMICs) or interventions. Each research question is addressed in turn with key findings highlighted alongside supporting in-depth narrative summaries and theoretical synthesis. Given suicidal behaviors and economic factors can be measured at the aggregate level (e.g., suicide rates, unemployment rates, aggregate government expenditure on welfare payments) or individual level (e.g., self-reported suicidal ideation, financial hardship, diagnoses), this is clarified where applicable.

## Results

### What Is the Role of Economic Factors Such as Un/Underemployment, Financial Hardship, Financial Wellbeing, Job Insecurity, and Economic Crisis on Suicidal Behaviors and Ideation?

#### Unemployment, Economic Crisis, Recession, and Suicidal Behavior and Ideation

##### Economic Crisis, Recession, and Unemployment–Aggregate Level

According to Durkheim (16), rapid social changes can cause “anomie” where societal norms are no longer acceptable, or accurately reflective of social reality, which increases the rate of suicides in the society/community (anomic suicides). Luo and colleagues (17) define economic crisis as “the state of affairs broken by sudden and severe economic recession” (p. 1139). The main characteristics of an economic recession are an increase in unemployment and a drop in gross domestic product (GDP) (17). Suicide mortality at the time of economic recession and crises has been the interest of numerous studies.

A notable body of research in the last decade has focused on the 2008 Global Financial Crisis (GFC), and typically compared the periods before and after the crisis, but also examined the association with unemployment at the time of crisis. A recent systematic review by Frasquilho and colleagues focused on multiple aspects to identify associations between recession, socioeconomic factors and mental health in the literature from 2004 to 2014 (18). Investigating the effects of pre-and post-recession changes in suicidal behaviors, they identified eight aggregate level studies using ecological study designs all focusing on the impact of the GFC. Studies were from Europe and Northern America and all except one analyzed suicide rates. In general, studies showed an increase in suicide rates after recession commencement, particularly for men and among the middle-aged. The only study analyzing suicide attempts, which was from Andalusia, Spain also showed a significant rise in hospital recorded suicide attempts after the recession onset (19).

A good example of a time-trend analysis of the impact of the 2008 GFC on suicide, not included in the above-mentioned review, included 54 countries: 27 in Europe, 18 in the Americas, eight in Asia and one in Africa (20). Their analysis assumed that excess suicides were caused by the onset of the GFC in 2008, therefore, excess suicides in 2009 were calculated using the trend line on 2000-2007 as the basis for expected suicides. They found 5,124 excess suicides for males: the increase was found for males in Europe (4.2%) and in the Americas (6.4%), but not in other (mostly Asian) countries. The largest increase was found for males aged 15–24 years in Europe and aged 45–64 in the Americas. There was no change for females in Europe, and the increase was smaller for females compared to males in the Americas. The authors also indicated that rises were associated with the magnitude of change in unemployment and were more prominent in countries with lower suicide rates before the crisis (20).

The systematic review by Frasquilho et al. (18), noted above, also identified studies analyzing correlations with the macroeconomic factors such as unemployment rate and GDP. They found 16 ecological studies in 2004 to 2014 showing strong associations between unemployment and suicide rates predominantly in European and North American countries covering varying time periods. A study by Norstrom and Gronqvist (21) covered the most countries (30 countries from the EU, North America and Australia) and involved the longest time period (1960-2012). They showed that the association between unemployment and suicide was strongest in the countries which had the least supportive unemployment protection (Eastern and Southern Europe). The association was significant for males in all country groups (grouped by strength of the welfare system) except Scandinavia, but for females it was significant only in Eastern Europe (i.e., lowest levels of protection). The interaction term capturing the possible excess effect of unemployment during the financial crisis was not significant.

Another systematic review covered a time period between 1992 and 2014 and identified 38 studies on the aggregate level focused on analyzing associations between macroeconomic factors (mainly unemployment rate and GDP) and suicide rates (22). They identified 31 studies that found positive associations (i.e., increased unemployment rate, decreased GDP associated with increased suicide rates), two studies that found no association, three that were inconclusive, and two that showed a negative association between economic recession and suicide rate.

A comprehensive analysis (23) aimed to improve understanding of the effect of unemployment on suicide rates by analyzing suicide mortality between 2000 and 2011, including other economic variables such as GDP, growth rate and inflation, using longitudinal modeling. Their methodology allowed for separate estimates to be made of excess suicides due to unemployment and due to the economic crisis (24). The 63 countries analyzed were categorized into four world geographic regions including the Americas, northern and western Europe, southern and eastern Europe and non-Americas and non-Europe. Only unemployment rate was associated with similar effects in the regions analyzed. The best fit model was the non-linear, 6-month time-lagged unemployment rate, displaying similar estimates for each world region. This means that rates of suicide tended to increase 6 months prior to unemployment rates rising, which might indicate the effect of job insecurity and work-related stress on suicide rates. Nevertheless, across all world regions between 2000 and 2011, 20–30% of suicides were related to unemployment. In 2007 and 2009, unemployment was associated with an estimate of 41,148 and 46,131 suicides respectively, suggesting that the recession was responsible for an additional 4,983 (unemployment related) suicides. This means unemployment was responsible for a 9-fold increase in suicides than those attributed to other impacts of the economic crisis, such as inflation (23). However, unemployment does not account for all the effects and impacts of the economic crisis. Recessions can also lead to potential cuts in public funding (i.e., fiscal austerity), inclusive of health care, job insecurity, lower income, debts, and bankruptcies which impact the lives of individuals and their families (24).

A recent international analysis further confirms the association between unemployment and suicide rates. One influential study, with a global coverage of 175 countries between 1991 and 2017, demonstrated a 1% increase in the unemployment rate globally is associated with a rise in male suicide rate by 1% relative to female (25). A stronger association of unemployment and male suicide rates is particularly evident in high income countries (4%). Comparisons by age groups showed that people aged 30–59 years were more impacted whereby a 1% increase in unemployment increased suicide rates by 2–3%. Their further analysis of GDP showed that an increase of the GDP per capita by every US$1,000 was associated with a decline in suicide rate by 2%. Interestingly a country-based analysis did not show any association between the GDP and suicide rate in Australia and the US (25). However, length of unemployment may be an important consideration (26).

In addition to analyzing unemployment rate, GDP per capita, or suicide rates throughout economic cycles, several studies also examined other macro-economic variables such as aggregate consumer behavior. For example, Korhonen et al. (27) created an economic hardship index based on the difference between habitual and actual consumption. Their panel data analysis of 15 OECD countries between 1960 and 2010 showed a relatively strong association between the increase in economic hardship index and increasing suicide rates after controlling for several aggregate level indicators, including the unemployment rate. The authors noted that a hardship index is a better explanatory variable than unemployment rate (27). A more recent Australian study (28) analyzed monthly data of suicide rates, unemployment, and the consumer sentiment index by gender from February 1990 until September 2018. This study is the first to analyze the link between suicide mortality and consumer sentiment (i.e., the perception and expectations of personal and wider economic conditions). Male suicide rates increased with a rise in unemployment rate but declined when consumer sentiment improved. Interestingly, suicide rates did not react to a decline in unemployment and to the worsening of consumer sentiment. The association was the opposite for females, where suicide rates increased significantly when consumer sentiment deteriorated and declined when unemployment rates dropped (28). The authors emphasized that Australian suicide prevention policies should target unemployment and financial problems as important risk factors, with special attention paid to men during major economic recessions.

In general, economic crisis, unemployment rate and other macroeconomic measures are associated with increased risk of suicidal behavior at the aggregated level. However, while ecological studies are useful for understanding changes on the aggregated level and generating hypotheses, there remain questions of causality (direct or indirect), as well as what other potential factors might be involved. Indeed, aggregated level studies are subject to an “ecological fallacy” and cannot explain associations at the individual level. More individual level studies are needed to provide further insight into the link between unemployment, financial problems and suicidal behavior and ideation.

##### Unemployment-Individual Level

Several systematic literature reviews and meta-analyses have focused on the links between unemployment and suicidal behaviors and ideation at the individual level. A recent meta-analysis examining the association between unemployment and suicidality (including suicide, suicide attempt and suicidal ideation) incorporated results from 54 studies across the world (published before April 2020), although mainly from Western countries, and to a lesser degree Asian and African based studies (29). The results showed a significant association between unemployment and suicide mortality [odds ratio (OR): 1.87, 95%CI: 1.40–2.50], suicide attempts (OR: 1.54, 95%CI: 1.26–1.89), and suicidal ideation (OR: 1.94, 95%CI: 1.61–2.34). However, the review included different study types, making comparisons difficult and thus could only describe associations.

The link between unemployment and suicidal behavior at the individual level is not clearcut. The most appropriate study designs for testing causality are cohort studies, which enable researchers to follow individuals over longer periods of time. Two systematic reviews and meta-analyses have specifically examined the unemployment-suicide relationship utilizing individual-level cohort studies (11, 30). A conceptual review and meta-analysis (11) aimed to add further clarity around social selection and social causation (Figure 1). Several of the included cohort studies demonstrated that unemployment is linked to suicide (11). The review (11) showed several cohort studies have tended to make an assumption of “social selection” and indeed their meta-analysis found that after adjusting for other factors such as mental health, the link between unemployment and suicide reduced; however, remained significant (RR: 1.15 95CI: 1.00–1.30). However, if mental disorders are considered as a mediator between unemployment status and suicide (i.e., “social causation”), then adjusting for mental disorders is methodologically flawed and may underestimate the impact of unemployment. A small number of cohort studies analyzing duration of unemployment, have shown that long-term unemployment is associated with a higher risk of suicide compared to short-term unemployment or to employed populations (30). The greatest risk for suicide was found within 5 years of unemployment as presented in another review by Milner and colleagues (30). However, mental health problems are not the only mediating factors; others include financial stress due to loss of income, and changes in health behaviors, among others (12).

It is important to consider that all studies included in the two meta-analyses by Milner et al. (11, 30) came from Scandinavian countries, which have comprehensive social welfare systems and thus there is potential for their support systems to mitigate the effect of short-term unemployment. Therefore, applicability of these results to other countries is debatable. Furthermore, comparisons across the studies are hindered by differences in the definitions of unemployment and suicidal behavior and ideation, study designs and statistical modeling (e.g., method and inclusion of confounding factors).

A more recent meta-analysis of longitudinal studies focused on the link between demographic factors, including employment status (defined by various available factors across studies such as occupation, type of employment, unemployment, skill level etc.), and suicidal behavior and ideation (31). They reported that employment status did increase the risk of suicide (RR: 1.41; 95CI: 1.05–1.90) and suicidal ideation (RR: 1.23; 95CI: 1.02–1.49), but not suicide attempt (OR: 1.12; 95CI: 0.74–1.70). However, their analysis grouped unemployed individuals with people with disabilities; therefore, it is not possible to distinguish the specific effect of unemployment due to economic reasons.

Unfortunately, most systematic reviews focus mainly on Western and high-income countries. One systematic review (32) focused on socio-economic factors and suicide (attempts) in low- and middle-income countries (LMICs) across Asia prior 2013. They identified 12 studies measuring the association between unemployment and suicidal behavior. While most studies did not find any association, three studies (from India, Indonesia, and Pakistan) found that people who died by suicide were more likely to be unemployed. A more recent large scale cohort study from Sri Lanka (33) also did not identify association between unemployment and suicidal behavior; however, they found that people from lower socioeconomic positions (e.g., daily wage laborers) had higher risk of suicidal behavior.

Gender differences at the individual level were only examined in the meta-analysis by Amiri (29). The findings indicated a significant association between increased odds of suicidality and unemployment in males (OR: 1.97, 95%CI: 1.44–2.70) and females (OR: 1.87, 95%CI: 1.48–2.37) with only a slight difference between sexes (29). For example, a recent study from New Zealand linking Census information of employment with the suicide mortality and hospitalization for intentional self-harm showed, after adjusting for confounders, unemployment was associated with suicide and self-harm similarly for men (adjusted OR: 1.48, 95% CI: 1.20–1.84 and adjusted OR: 1.55, 95% CI: 1.45–1.68, respectively) and women (adjusted OR: 1.39, 95% CI: 1.13–1.37 and adjusted OR: 1.39, 95% CI: 1.13–1.37, respectively) (34). Nevertheless, some recent results contradict these findings. A US study utilizing data from the National Longitudinal Mortality Study including 1.5 million people, identified that sex was a moderator in the association between unemployment (looking for work) and suicide (35). More specifically, the association was stronger for women (adjusted RR: 2.99, 95% CI: 2.05–4.37) compared to men (adjusted RR: 1.39, 95% CI: 1.13–1.37) after adjusting for demographic variables. An Australian study, utilizing the National Coroner's Information System, showed that unemployed/economically inactive males had over four times the risk of suicide compared to the employed, which was over eight times the risk for females (36). However, a further analysis of the potential impact of the GFC on suicide showed a significant increase in suicides in economically inactive/unemployed males (22% in 2008, *p* < 0.001) and females (12% in 2007, *p* < 0.001). Nevertheless, suicide also increased among economically active males (7% rise in 2007 *p* = 0.003), but not among employed females.

#### Financial Problems and Suicidality

Economic problems such as unemployment and underemployment are highly interrelated with financial problems such as debt and financial strain. It cannot be assumed that just one in isolation leads to suicidality, but rather a combination is likely. There are further complexities when considering the issue of definitions and terminology. French and Vigne (37) define “*financial strain* as anxiety, worry or feelings of not coping created by economic or financial events. This condition is therefore synonymous with ‘financial/economic hardship,' ‘financial/economic stress,' ‘financial difficulties' or ‘inability to cope financially.' We regard economic problems such as unemployment, poverty, arrears, debt or even over-indebtedness as necessary but insufficient explanatory factors for financial strain.” (p. 150). Although there are some aggregate level studies [e.g., (27, 38)] showing a link between economic hardship based on consumption and suicide, the majority of research analyzes individual level links.

A systematic review and meta-analysis (39) examined unsecured debt (e.g., credit) and suicide across nine studies, and found a significant association between debt and suicide (OR: 7.9, 95% CI: 5.21–12.0) and suicidal behaviors (pooled OR: 5.76, 95% CI: 2.97–11.18). Another systematic review focusing on indebtedness and its health impacts referred to five studies analyzing debt and suicidality and concluded that people with unmet loan payments were more likely to experience suicidal ideation (40). Interestingly, a US study found that people who were admitted to the trauma center with a suicide attempt had significantly higher odds for becoming bankrupt in the following 2 years compared to those admitted with an accident, after adjusting for several confounders (OR: 2.10, 95% CI: 1.29–3.42) (41). This finding was stronger for females. Odds of personal bankruptcy in the 2 years before a suicide attempt were somewhat weaker (OR: 1.68, 95% CI: 1.06–2.67). The results revealed that filing for bankruptcy is not an isolated event and does not reflect the end or the beginning of financial hardship and suicidality (41).

Other studies further show the interrelatedness of financial problems with unemployment and other factors. For example, in a recent US cohort study Elbogen et al. (42) found that cumulative financial strain, which encompassed financial debt/crisis, unemployment, past homelessness, and low-income, was predictive of suicide attempts (OR: 1.53, 95% CI: 1.32–1.77) and suicidal ideation (OR: 1.44, 95% CI: 1.33–1.55) between Waves 1 (2001–2002) and 2 (2004–2005) after controlling for demographic and clinical covariates. Moreover, when examining these factors independently, at Wave 1 financial debt/crisis and unemployment were predictive of suicide attempts and suicidal ideation between the two waves (42).

Recent studies analyzing various aspects of financial strain in South Korea utilized the Korean Welfare Panel Study with over 10,000 participants. Kim and You (43) analyzed late bill payments and after adjusting for sociodemographic variables and self-reported depressive symptoms, suicide attempts were significantly and positively associated with overdue payments. More specifically, people with late bill payments had increased odds of suicide attempts rising with the number of late payments (one - OR: 5.46; 95% CI: 1.82–16.39, two or more–OR: 7.44 95% CI: 2.89–19.20) compared with those without late payments (43). Furthermore, having one late payment was not significantly associated with suicidal ideation, but having two or more late payments increased the odds of suicidal ideation significantly (OR: 2.11, 95% CI: 1.22–3.65) (43). Another analysis examined seven waves from the same dataset (44). Financial hardship was measured as a composite of multiple questions (including difficulties in paying for rent, utilities, healthy food, use of medical services, and other credit problems) and change over time, and was categorized as no hardship, resolved, emergent and persistent over 2 years (44). The results showed a significant association between financial hardship and suicidal ideation. In particular, after adjusting for confounding factors, emergent and persistent hardship were each associated with suicidal ideation for both genders and all age groups. Additionally, for resolved hardships, the association with suicidal ideation was still significant for men and women aged 65 years and older (44).

#### COVID-19 Pandemic, Economic Factors, and Suicidality

The COVID-19 pandemic has led to increased unemployment, financial strain, and economic downturn. Indeed, financial insecurity as measured by a variety of indices (e.g., market volatility, subjective uncertainty, forecaster disagreement etc.) has peaked rapidly at unprecedented rates (45). These economic circumstances may further lead to a rise of mental health problems and suicidal behavior (46). At the early stages of the pandemic, several expert opinion pieces (47–49) and predictions emerged (50). All refer to the potential impact of economic conditions on the aggregate and individual level, which are likely to lead to an increase in suicidal behavior and ideation. An ecological study investigated the expected effects of the COVID-19 related economic turmoil by modeling predicted suicide rates in 38 OECD countries in 2000-2017, to examine the association with unemployment (46). The results suggested that unemployment was significantly associated with higher suicide rates in men aged 15–64 years, particularly for men aged 40–64 years. This relationship was much weaker for women, with the unemployment-suicide relationship significant for girls and women aged 15–24 and 35–74 only (46). However, despite the authors' noting the relevance of their modeling in the context of the COVID-19 pandemic, they did not make any attempt to predict future changes in suicide rates. McIntyre and Lee (50) did attempt to make predictions in a Canadian study by analyzing different scenarios in relation to the change of unemployment. However, this approach is fraught with methodological challenges, considering the multiple factors impacting suicidal behaviors, with some potentially having a protective effect at the time of crisis (e.g., togetherness, resilience, and others) (51). Indeed, in the early stage of the pandemic suicide rates have not increased (52, 53).

A few longitudinal studies have also analyzed economic stressors at the time of COVID-19 and suicidal thoughts. For example, a Canadian repeated cross-sectional study investigated the prevalence of self-reported suicidal ideation in a nationally representative sample during the COVID-19 pandemic at three time periods between 2020 and 2021 (54). The results indicated the prevalence of suicidal ideation is increasing over the course of the pandemic. Analysis of COVID-19 related concerns showed that after adjustment for sociodemographic factors, individuals who were experiencing financial stressors, such as concerns about debt and paying bills, had increased risk of suicidal ideation (OR: 2.48, 95% CI: 1.97–3.13). Furthermore, worries about job loss were also associated with increased odds of suicidal ideation (OR: 2.61, 95% CI: 2.07–3.29) (54). A longitudinal online study from the UK over two timepoints in May and September 2020, examined whether COVID-19 related financial stress and social isolation were associated with suicidal ideation and behavior in a small sample (*n* = 370) (55). Financial stress deemed by the respondent as COVID-19 related at time point 1 was significantly associated with suicidal ideation and behavior at time 2, (*p* = 0.01). Depression and loneliness were also found to significantly mediate the relationship between financial stress and suicidal ideation and behavior at time point 2 (55).

As the impact of the COVID-19 pandemic continues to unfold, it is important that ongoing and high-quality surveillance of suicidal behavior and ideation continues (52). This is critical for determining the overall impact of COVID-19 on suicidal behaviors, and in particular, the economic impact of such an unprecedented pandemic on a global scale. Based upon the literature in this review it appears that suicide rates have not increased in the early stages of the pandemic, and may in fact, have decreased (52). However, it does appear that financial concerns attributed to the pandemic may contribute to later suicidal ideation and distress which may have an ongoing impact on suicidal behaviors in the future.

### What Available Evidence Is There for the Effectiveness of Interventions Addressing Economic Factors in Reducing Suicidal Behaviors and?

#### The Protective Role of Policy and Government-Based Interventions

Government policies and expenditure directed toward mitigating the impact of harmful economic circumstances (e.g., unemployment) may not be traditionally regarded as suicide prevention interventions. However, given the associations described above and important theoretical conceptualizations it is conceivable that such activities may reduce suicidal behaviors and ideation, as well as improve overall mental health and wellbeing (7, 8, 56). A recent systematic literature review of studies published until October 2018 sought to determine whether government level responses to economic factors ameliorated the relationship between unemployment and suicide (57). Only six ecological studies examining unemployment policy (e.g., benefits, protection legislation) on suicide rates were identified. Each study spanned several years and multiple high-income countries/states. Overall, the authors concluded there was evidence to suggest government unemployment supports were associated with a reduction in suicide rates (57). This has important implications for suicide prevention. For example, two of the included studies (58, 59) examined the impact of active labor market programs (ALMPS) across an overlapping cross-national sample in the European Union. ALMPS are defined by the Organization for Economic Co-operation and Development (OECD) as all social expenditure, besides education, with the intent of improving chances of gainful employment or an increase in earning capacity (60). Both studies found that for every increase in unit of spending on ALMPs there was an associated decrease in suicides (albeit only small 0.026–0.038%). However, neither study found any mitigating impact of employment benefit payments by either total aggregate spending (59) or income replacement per unemployed person (58). In contrast, three further studies identified in the review found higher unemployment benefits were associated with significant decreases in suicide rates (61), particularly in men (21, 62). Fiscal austerity and *reduced* government spending was associated with a short (1.38%), medium (2.42%), and long-term (3.32%) rise in suicide rates in older aged men (62). In these studies, the operationalization of employment benefits was more encompassing and attempted to capture overall “generosity” of benefit. For instance, maximum rate multiplied by maximum duration of eligibility (61), gross replacement rate (62), as well as the incorporation of other characteristics such as wait times and qualifying conditions (21). The final study included in the review included high income countries within the OECD (18 European countries, Japan, and Republic of Korea) across 1994 to 2010 (63). This study investigated the impact of employment protection legislation (against unfair dismissal) in *younger* adults (25–34 years) and found that for those with regular work contracts there was a significant protective effect of legislation regardless of sex, whereas for temporary workers effects were only observed in men aged 30–34 years of age. This was also found in older aged men (62). Overall, the systematic review (57) noted that further research was needed and would benefit from more rigorous testing (e.g., cohort designs), to investigate impacts at the individual level (e.g., qualitative designs), as well as to evaluate the possible impact on suicide attempts or self-harm.

More recently, several studies published after the systematic review (57), have also investigated the impact of government-based interventions and overall suicide rates. A recent ecological study in Italy examined the relationship between rates of unemployment and suicide in men and women separately from 1990 to 2014, with a focus on the recession, and investigated whether ALMPs moderated this relationship (64). Average ALMP spending per head did appear to moderate the unemployment-suicide relationship in men aged 45–54 who were in a central region in Italy, whereby a 1% increase in ALMP spending was correlated with a 0.45% decrease in suicide rates among men in this subgroup (64). No significant impact was noted for women in this age group and region, or for people in any other age groups located in or outside of central Italy (64). The authors suggest that a lack of adequate funding may have influenced the absence of widespread findings across subgroups, as spending was far below minimums reported in other studies [US$125 per head in the current study vs. US$190 suggested by (59)].

Regarding the *accessibility* of unemployment benefits/insurance, rates of insurance recipiency (as a measure of eligibility and implementation, not total benefit spending ratio or benefit duration) were deemed potentially protective at a population level for those with highest rates of suicide such as men and those aged 45–64 years in all states of the US from 2000 to 2015 although findings were not significant (65). In another US study, an increase in the mandated minimum wage by US$1 reduced suicide by 6% in those with low education (aged 18–64) whereas there was no impact for those with college degrees even when adjusting for age, gender and ethnicity, using data from all states in USA (1990–1995) (66). This relationship was stronger in periods of high unemployment and attenuated in periods of low unemployment, with the authors concluding that policies aiming to improve economic circumstance of those in lower socioeconomic positions in particular, can have a protective effect on suicide (66).

It appears that despite the well-established connection between economic factors and recession with suicidal behaviors, there is a comparatively small body of research investigating the protective role of government policy interventions with regards to suicide prevention, especially when considering suicide attempts, self-harm, and suicidal ideation. However, as noted by Shand et al. (57) suicide is an “extreme” outcome from unemployment. Other literature reviews have noted the beneficial impact of ALMP initiatives and benefit payments/social protection spending on physical and mental wellbeing, including depressive symptoms [see (67, 68) for review]. Unfortunately, this may be less protective than actual employment for men (69) or for those with insecure jobs (70). In contrast to suicidal behaviors, these findings for mental wellbeing were demonstrated mostly at the individual level (e.g., self-reported symptoms of depression, anxiety, or poor wellbeing).

Furthermore, given the complex and compounding associations with other prominent risk factors for suicidal behaviors, and the likely co-occurring role of social causation and social selection described earlier, it has been suggested that government policies to minimize the harmful effects of alcohol and other drugs, reduce homelessness, promote social inclusion, facilitate equitable access to primary (mental) health care, support low-income families, and encourage the responsible media reporting of suicidal behaviors [see (7, 8)], may be additional (and often established) primary preventative measures that may also ameliorate the association between economic factors and suicide. According to social causation, addressing economic factors has the potential of reducing mental health difficulties and by extension suicidal behaviors, and according to social selection, may prevent an intensification of already present risk factors for suicidal behaviors. Given these models' likely overlap (7) it appears policy level interventions may be beneficial in protecting against suicidal behaviors and distress; however, more research is required.

#### Individual Level Interventions Addressing Employment and Personal Financial Circumstances

In addition to government policies, there is the potential to provide tailored interventions for economic advice and assistance that may aid in the prevention of suicidal behaviors at the individual level. Research, however, is sorely lacking. A small-scale feasibility study of a randomized control trial in the UK used a mixed methods design to examine the feasibility and acceptability of an intervention (Help for People with money, employment, or housing problems “HOPE” service) (71). The intervention provided psychosocial support for individuals who presented to the emergency department following self-harm or acute distress due to (accumulating) employment, financial, or welfare issues (71). The novel and assertive intervention was developed in recognition of the vast difficulties people have in navigating the employment benefits and social welfare system, application processes, delays, and meeting eligibility requirements. Even though these policies and benefits are designed to assist, the administrative processes have been cited as a source of huge stress in the lead up to self-harm emergency presentations, among others (72, 73). In the intervention group (*n* = 13), participants received a series of one-on-one tailored financial assistance sessions (e.g., interpretation of official documentation, benefits advice, connection with community resources and mental health care) supplemented with motivational interviewing designed to resolve ambivalence, boost independence, decision-making skills, and confidence when addressing their financial problems. Sessions were mainly conducted in the home, however, also involved travel to debt advice agencies. In the control group (*n* = 9), participants were signposted to support organizations. Qualitative feedback from participants (*n* = 19 randomized 2:1) and workers providing the intervention suggest there was benefit to the program, including assistance with resolving financial difficulties (71). However, being a feasibility trial, it is necessary for future research to determine actual effectiveness of the intervention as compared to the control group.

Given a lack of information on suicidal behaviors, we broadened our focus to examine literature that has investigated financially focused interventions that aim to improve mental health and wellbeing. A recent systematic review of community interventions (68) examined the effectiveness of interventions aimed at acute financial uncertainty, such as financial strain, job loss, and debt, in improving mental health outcomes. Searches concluded in August 2019 and studies were included if they reported mental health outcomes in working age adults (18–64 years) in high income countries and used experimental, quasi-experimental or observational designs. A total of 15 studies met the inclusion criteria. Two studies evaluated telephone debt advice interventions (74, 75). One study in the UK found no significant changes in anxiety at the 20 week follow up, and due to a high attrition rate, the 12-month follow up was not completed (74). The second study in the US found only small improvements in overall health, which included stress, however mental health was not assessed independently (75). A further seven studies examined the effectiveness of welfare advice services co-located within healthcare settings and found mixed results. One examined food insecurity interventions (e.g., food banks), and two examined gatekeeper signposting and referring to community supports (68). Overall, the authors noted that review findings were limited by poor quality design (e.g., small, uncontrolled studies), yet interventions appeared useful in improving financial distress. However, it was not clear as to the effectiveness on mental health outcomes (68).

An earlier systematic literature review focused exclusively on randomized control trials investigating interventions targeting debt and unemployment, including debt advice, gatekeeper training, job skills training and others (76). Studies were excluded if participants had serious mental illness, were not of working age, were part of a specific group (e.g., single mothers), or were focused on rehabilitation into the workforce for those with serious physical or mental health problems. Despite overlap in the search period, only two studies overlapped with the previous review by McGrath et al. (68) (one assessing debt advice hotline, and one assessing a group job skills training intervention). This review found, based on multiple trials, intensive 1-to-2-week job skills and self-efficacy training (“job clubs”) were effective in reducing depression for up to 2 years. However, results were less clear for unemployment. Furthermore, cognitive-behavioral therapy for long-term unemployed people and those in lower socioeconomic groups were effective in reducing symptoms of depression and improving re-employment. In this review, only one study was identified that examined the effectiveness of a debt advice hotline [overlapping with (68)] as well as one trial each for various other psychological interventions (e.g., imagery, journaling) and thus evidence was deemed limited for these approaches (76). Unfortunately, this review was limited by its strict exclusion criteria which meant that studies did not include participants who may be at particular risk of unemployment of financial hardship and also suicide (76).

Altogether, these reviews demonstrate the effectiveness of financial and employment-based interventions on reducing mental health symptoms, particularly depressive symptoms. Given associations between mental health and suicidal behaviors this could have implications for suicide prevention (76). These reviews provide inconclusive evidence as to the effectiveness of debt advice interventions (e.g., helplines) and trials had difficulties with recruitment and attrition overall.

#### Interventions Implemented During and in Response to COVID-19

There has been considerable and well justified concern regarding the unfolding impact of the COVID-19 pandemic on both suicidal behaviors and economic crisis, including unemployment. As a result, governments around the world have introduced unprecedented social welfare packages. As described earlier, policy-based employment interventions may have beneficial outcomes on suicide rates, including during periods of economic recession; however, this is not clear for suicide attempts and self-harm (57). Furthermore, most evidence across both levels were for ALMPs and employment focused interventions or policies which do not apply to the COVID-19 pandemic where whole industries were affected (e.g., “gig” economy, hospitality, tourism, transport) and opportunities for (re)employment were necessarily limited due to health restrictions. Therefore, government activities have included raising expenditure on employment benefits among other stimulus measures. As described earlier, overall generosity of benefits has been linked to reduced suicide at the aggregate level (57) and it remains to be seen what impact this has on other suicidal behaviors and at the individual level. Nevertheless, suicide rates did not rise in the initial stages of the COVID-19 pandemic (52), and employment benefits and social welfare payments (among others) have been theorized as possibly underlying mechanisms explaining this finding (77). Recent research examined data derived from helpline calls in 19 countries, focusing on the first and subsequent waves of the COVID-19 pandemic. The relationship between call types, income support offered, and the lockdown policies in place in specific countries were investigated (78). Overall, the results suggested that helpline calls increased and peaked 6 weeks after the start of the pandemic, with an increase in calls related to fear and loneliness. However, there was a decline in calls related to suicidal ideation. The latter may have been attributed to a shift of focus to the concern of others, or their fears of COVID-19 infection (78). Measured by an income support index, data from two of the largest helpline samples in France and Germany were further analyzed. Results indicated an increase in infection rates and more generous income support were significantly associated with a lower number of suicide-related calls in France (*p* = 0.004) and Germany (*p* < 0.001) and it was suggested that for individuals affected economically by the pandemic, the income support provided may have helped to reduce mental distress. However, there is a need for ongoing research to provide a deeper understanding of financially focused intervention or policy during COVID-19 at the individual level.

## Discussion

This review sought to synthesize recently (since 2010) published information on the role of economic factors at the individual and aggregate level, including un/underemployment, financial hardship, job insecurity and economic recession on suicidal behaviors and ideation, as well as the effectiveness of interventions addressing these factors on reducing suicidal behaviors. The impact of COVID-19 was highlighted where available.

Based upon the current review and previous others (7–9, 11, 30), it is clear that periods of economic recession and unemployment are associated with an increased risk of suicidal behavior at the aggregate and individual level. Furthermore, financial problems such as debt and financial strain are associated with an increased risk of suicidal behavior and ideation at the individual level. While these relationships are complex and the directionality of association is not clear cut, the (interrelated and overlapping) concepts of social selection and social causation provide two theoretical frameworks for how socioeconomic circumstances may influence or be influenced by mental health and contribute to risk for suicidal behavior and ideation (see Figure 1). Furthermore, several prominent theories have attempted to determine the genesis of an individual's suicidal ideation and the mechanisms or constructs underlying the transition from ideation to intent, and ultimately, to self-harm or suicide (79–81). As depicted in our figure, economic circumstances (at the individual and aggregate level) likely influence and are influenced by these mechanisms, such as mental health, social isolation, and connectedness (80, 81). For example, in the integrated motivational-volitional model of suicidal behavior (79), economic disadvantage and recession are acknowledged as important contextual events in the pre-motivational phase of suicidal behavior. However, future research is warranted to determine how further aspects of financial hardship relate to defeat, humiliation, and entrapment as well as socially prescribed perfectionism and negative social comparison, all of which are central to the motivational phase and emergence of suicidal ideation (79).

It is presently unclear what impact economic factors, during or as a result of the COVID-19 pandemic, may have on suicidal behavior and ideation. Nevertheless, it appears that suicide rates have not increased in the early stages of the global pandemic (52). A reduction in suicides in the initial period following large scale disasters is often referred to as the “honeymoon” period where individuals and communities as brought together by their experience of the disaster, and this has been linked to the COVID-19 pandemic (77). Given most data so far was from earlier stages of the pandemic and economic fallout continues to unfold, there is an important need for ongoing research. Especially as people return to work, leaving others behind (26), and as countries reduce their employment benefits to pre-pandemic levels (52, 77).

This review also highlights that unemployment benefits, employment protection legislation, minimum wage and active labor market programs may reduce suicide at the population level, particularly for men [see (57)]. However, the research is somewhat limited and mixed, and it is not clear what impact they have at the individual level. Further, there were no identified studies or reviews investigating outcomes directly in relation to suicide attempts, self-harm, or suicidal ideation. Studies were largely ecological as this type of policy level intervention does not lend itself easily to more robust research designs and is limited to the aggregate level. Overall, more research is required, particularly in relation to individual level outcomes, and the cost-effectiveness of such policy interventions. There was also a lack of evidence as to the effectiveness of tailored financial focused suicide prevention interventions at either the individual or aggregate level. However, there was some evidence that these interventions (e.g., “job club” groups) may improve depressive symptoms over time, which could have implications for suicide prevention by extension [see (76)]. This general lack of research extends to effectiveness of interventions during the COVID-19 pandemic, and various complicating factors make conclusions difficult at this time. Nevertheless, expert opinion and discussion (52, 77) suggests the unprecedented social welfare measures implemented by governments internationally may have had a protective effect against suicidal behaviors.

The complex web of associations between economic factors and other prominent risk factors for suicidal behaviors and ideation (e.g., mental health, substance use) warrants establishing or enhancing responsive, effective, and compassionate interventions that are equitable and accessible in addressing these factors at the individual and aggregate level [e.g., (7)]. Based upon the findings of the current review several recommendations can be made that may serve to mitigate risk at the aggregate and individual level as per the (overlapping) social selection and social causation models, although it is important to be mindful of the methodological issues with existing research regarding causality and endogeneity. For example, higher and more generous welfare payments (i.e., accessible, timely) should be established or maintained as they may have a protective effect against suicide at the aggregate level (8, 57), particularly for those in more vulnerable or at-risk groups (e.g., lower education, youth, men during periods of low unemployment, those with unstable housing, vulnerable industries). This may also be particularly relevant during periods of economic crisis and recession, where the complex and accumulative impact of financial stress in contributing to mental health problems and suicidal behaviors may in turn create a demand on health and mental health services which would be under resourced in times of reduced government spending and have disproportionate impact on those in worse economic positions during the recession (8). Cross-sectoral collaboration may be particularly important in developing suicide prevention policies that adequately and equitably address issues of mental health and substance use, as well as issues of economic factors and social welfare, housing etc. Additionally, the development and evaluation of individual level support services based on promising evidence from small-scale international studies [e.g., HOPE; (71)] should be considered. However, people with lived and living experience of suicide and financial hardship (unemployment, debt, recipient of benefits) should engage in co-designing these interventions to maximize motivation (and minimize attrition). It may also be important that staff working in employment, welfare, or other socioeconomic institutions (e.g., banks) receive regular suicide prevention training (8). Ideally, these programs would also be co-designed collaboratively with those who have lived and living experience of suicidality and financial hardship, and would be accredited per national standards. These programs must also be designed to achieve certain core competencies published in the literature and be evidence-informed (82). Finally, promoting awareness of free financial services (e.g., financial counseling, debt, and gambling helplines, online “self-help” resources) may increase general levels of financial literacy and connect individuals with supports within the community to respond quickly to personal economic hardship before problems escalate, and may be particularly useful as an early intervention (e.g., for young people). Indeed, future research would benefit from investigating the protective role of financial literacy and financial wellbeing, financial resilience, and/or financial self-efficacy on suicidal behaviors and ideation.

### Methodological Considerations

There are several points to consider when interpreting the findings from this review. Studies displayed substantial variability in the different time frames examined and the definitions or operationalization of economic constructs used (e.g., unemployment, underemployment, financial hardship), with several studies not providing a definition at all. This poses a challenge to integrating and generalizing findings. Nevertheless, the current review provides an overview of the current state of the field and identifies potential areas where more research is needed, particularly in relation to interventions at the population and individual level and advancing theoretical understandings with relation to non-fatal suicidal behaviors and ideation. Furthermore, it is likely that there may be some publication bias across studies, particularly at the ecological level, whereby those that found an association may have been more likely to be published. There was also an overall lack of research identified from low-and-middle income countries (LMIC). This is important as the majority of the world's population reside in these countries, which is also where the vast majority of global suicides occur (1). There are likely differences in the societal and cultural impact of economic factors (e.g., unemployment) on suicidal behaviors, as well as variation in the needs and capacity of governments and organizations in responding to these impacts. The few systematic reviews and cohort studies in LMICs across Asia (32, 33) suggest the associations between unemployment and suicidal behavior are less clear cut.

As may be expected, there was also a lack of research investigating interventions and protective factors aimed at addressing economic circumstances and suicidal behaviors and ideation. Of the studies that were identified, many were of low quality or small sample size and had issues with attrition/drop-out. This is important as the high dropout rates may suggest these types of financially focused interventions are not acceptable to financially stressed individuals. For example, it may be that interventions based on providing information do not account for the complex interrelated and intersecting difficulties that serve as reinforcing barriers. Alternatively, perhaps financial stress, and related circumstances, makes it more difficult for people to engage in interventions. Therefore, interventions such as HOPE (71) may be useful in attempting to provide additional motivational or psychological components. Based on the available evidence it is not possible to know at this stage. This lack of research was most noted at the individual level. There is a need for more studies that examine the impact of individual and modifiable protective factors on suicidal behaviors and ideation (e.g., financial wellbeing, resilience), including in situations of long-term unemployment or economic recession.

### Strengths and Limitations of the Current Review

Given our review focuses on various types of economic factors (recession, unemployment, underemployment, financial strain, debt, etc.) across the spectrum of suicidal behaviors, and across multiple levels, there was an imbalance in the extent of literature available for each. Therefore, we conducted a selective review to integrate information across fields of research and practice to provide fresh insight into the role of economic factors on suicidal behaviors and ideation, and possible effective interventions. This approach may have introduced some bias into findings. Nevertheless, we did focus on synthesizing information from systematic reviews and meta-analyses, high-quality cohort studies, or studies utilizing multiple years and countries. Findings were also discussed in relation to prominent theoretical models. Incorporating economic circumstances and traditional social theories (Figure 1) into individual level theoretical conceptualizations of suicidal behaviors and ideation (79–81) promotes greater understanding of suicidality from different perspectives (psychiatric, public health, economic disadvantage). This will facilitate and provide directions for a more streamlined approach to prevention opportunities.

## Conclusion

This review examined the role or association between economic factors (unemployment, financial hardship, job insecurity etc.) and suicidal behaviors and ideation. The review also examined the effectiveness of interventions at the government and individual level. Findings confirmed that economic circumstances are an important determinant of suicidal behaviors. Altogether, there was a comparatively smaller body of research examining the protective impact of government level policies and individual focused interventions on suicidal behaviors and ideation. However, it appears that policy level interventions aimed at alleviating financial stress during periods of increased unemployment may be beneficial in preventing suicide at the aggregate level, although based on existing study designs causality is difficult to determine. Our recommendations for future research to co-develop and evaluate new financial services with respect to impact on suicidal behaviors and ideation, evaluate the impact and cost-effectiveness of existing services and policy level interventions, as well as better determine the role of economic circumstances in relation to theoretical conceptualizations such as ideation to action frameworks and in Figure 1 will ensure a clearer understanding of the role of economic factors on fatal and non-fatal suicidal behaviors and ideation and assist in guiding the development of effective targeted interventions at both the individual and government level.

## Author Contributions

Conceptualization: KK, SM, VR, and JH. Data curation and formal analysis-synthesis of results: AT, SM, and KK. Funding acquisition: KK, SM, and VR. Supervision: KK. Writing–original draft: SM and KK. Writing–review and editing: JH, VR, and AT. All authors contributed to the article and approved the submitted version.

## Funding

Funding was received by Suicide Prevention Australia.

## Conflict of Interest

The authors declare that the research was conducted in the absence of any commercial or financial relationships that could be construed as a potential conflict of interest.

## Publisher's Note

All claims expressed in this article are solely those of the authors and do not necessarily represent those of their affiliated organizations, or those of the publisher, the editors and the reviewers. Any product that may be evaluated in this article, or claim that may be made by its manufacturer, is not guaranteed or endorsed by the publisher.
